# Regulatory T Cells in Pregnancy Adverse Outcomes: A Systematic Review and Meta-Analysis

**DOI:** 10.3389/fimmu.2021.737862

**Published:** 2021-10-29

**Authors:** Samantha Green, Marina Politis, Kathrine S. Rallis, Alba Saenz de Villaverde Cortabarria, Athina Efthymiou, Nicoleta Mureanu, Kathryn V. Dalrymple, Cristiano Scottà, Giovanna Lombardi, Rachel M. Tribe, Kypros H. Nicolaides, Panicos Shangaris

**Affiliations:** ^1^ University of Aberdeen School of Medicine and Dentistry, University of Aberdeen, Aberdeen, United Kingdom; ^2^ Undergraduate Medical School, University of Glasgow, Glasgow, United Kingdom; ^3^ Barts and The London School of Medicine and Dentistry, Queen Mary University of London, London, United Kingdom; ^4^ College of Medicine and Veterinary Science, The University of Edinburgh, Edinburgh, United Kingdom; ^5^ Department of Women and Children’s Health, School of Life Course Sciences, Faculty of Life Sciences and Medicine King’s College London, London, United Kingdom; ^6^ School of Immunology & Microbial Sciences, Faculty of Life Sciences & Medicine, King’s College London, London, United Kingdom

**Keywords:** regulatory T cells (Tregs), pregnancy, high blood pressure (hypertension), pre-eclampsia, pre-term birth (PTB), pregnancy adverse outcomes (PAO)

## Abstract

**Background:**

Several studies report the role of Regulatory T-cells (Tregs) in the pathophysiology of pregnancy adverse outcomes.

**Objective:**

The aim of this systematic review and meta-analysis was to determine whether there is an association between regulatory T cell levels and pregnancy adverse outcomes (PAOs), including pre-eclampsia and preterm birth (PTB).

**Method:**

Literature searches were conducted in PubMed/MEDLINE, Embase, and Cochrane CENTRAL databases. Inclusion criteria were original articles (clinical trials, case-control studies and cohort studies) comparing Tregs, sampled from the decidua or maternal blood, in healthy pregnant women *versus* women with pre-eclampsia or PTB. The outcome was standardised mean difference (SMD) in Treg numbers. The tau-squared (Tau²), inconsistency index (I²), and chi-squared (χ²) test quantified heterogeneity among different studies. Analyses were performed in RevMan software V.5.4.0 for Mac using a random-effects model with outcome data reported with 95% confidence intervals (CI). This study was prospectively registered with PROSPERO (CRD42020205469). PRISMA guidelines were followed.

**Results:**

From 4,085 unique studies identified, 36 were included in qualitative synthesis, and 34 were included in quantitative synthesis (meta-analysis). In total, there were 1,783 participants in these studies: healthy controls=964, pre-eclampsia=759, PTB=60. Thirty-two studies compared Tregs in healthy pregnant women and women with pre-eclampsia, and 30 of these sampled Tregs from peripheral blood showing significantly higher Treg numbers in healthy pregnancies (SMD; 1.46; 95% CI, 1.03–1.88; I²=92%). Four studies sampled Tregs from the maternal decidua showing higher Tregs in healthy pregnancies (SMD, 0.76; 95% CI, -0.13–1.65; I²=84%). No difference was found in the number of Tregs between early *versus* late pre-eclampsia (SMD,-1.17; 95% CI, -2.79–0.44; I²=94%). For PTB, two studies compared Tregs sampled from the peripheral blood with a tendency for higher Tregs in healthy pregnancies but this did not reach significance (SMD, 2.18; 95% CI, -1.34–5.70; I²=96%). Subcohort analysis using Treg analysis (flow cytometry *vs.* qPCR *vs.* immunofluorescence tissue staining) showed similar associations.

**Conclusion:**

Lower Tregs in pregnancy, sampled from the maternal peripheral blood, are associated with pre-eclampsia. There is a need for further studies to confirm a relationship between low Tregs and PTB. As the precise mechanisms by which Tregs may mediate pre-eclampsia and PTB remain unclear, further fundamental research is necessary to elucidate the underlying processes and highlight the causative link.

**Systematic Review Registration:**

PROSPERO, identifier CRD42020205469.

## Introduction

### Preterm Birth

Preterm birth (PTB) is defined by the World Health Organization (WHO) as birth prior to 37 weeks of gestation (1), further subdivided into extreme, very and moderate-to-late preterm occurring prior to 28 weeks, 28 to 32 weeks, and 32 to 37 weeks respectively. PTB has become the leading cause of perinatal morbidity and mortality in developed countries (2) and despite an overall decline in perinatal mortality, preterm infants face increased short-term morbidity and long-term neurodevelopmental, respiratory, and gastrointestinal complications (2).

The complex heterogeneity of PTB is due to its varying aetiology and pathogenesis, which result in idiopathic premature activation of the labour process, with or without pathological insults (2). Approximately 50% of PTBs are due to preterm labour (PTL), uterine contractions before 37 weeks’ gestation that may or may not progress to delivery (i.e., PTB), with intact membranes or preterm premature rupture of membranes (PPROM, 25%) (3). Up to 40% of these cases are due to intrauterine infection. Other causes include inflammation, vascular disease, uterine overdistension, placental abruption or hormonal disruptions (4–6). 25% involve induced labours or caesarean deliveries as a result of maternal or fetal indications (7). Due to effects on placental blood supply and intrauterine growth, pre-eclampsia is a prime example of such an indication, accounting for up to 20% of PTBs (8).

### Pre-eclampsia

Pre-eclampsia affects 3-5% of pregnancies, with incidence increasing due to a higher prevalence of risk factors including maternal obesity, older maternal age and diabetes mellitus (9). Pre-eclampsia is diagnosed in the presence of hypertension after 20 weeks’ gestation accompanied by maternal acute kidney injury, liver dysfunction, neurological symptoms, haemolysis or thrombocytopenia, or fetal growth restriction (10). Further risk factors include first pregnancy, hypertensive disease in previous pregnancies and co-morbidities including autoimmune and renal disease. Proposed pathways of pre-eclampsia suggest immunological factors of genetic and environmental origin are involved in the pathogenesis (11).

Early and late-onset pre-eclampsia, defined as onset before 34 weeks of gestation and at or after 34 weeks respectively. These are important to differentiate as different pathogenic mechanisms and outcomes are implicated – whilst shallow trophoblast invasion is common in early onset type (12), exaggerated inflammatory responses may play a role in the development of late-onset disease (12). Furthermore, aspirin treatment prevents early onset pre-eclampsia but not late onset disease (13). Thus, it is important to compare Treg numbers between these subtypes as they may represent different disease entities (12).

Clinical diagnosis requires proteinuria, new-onset hypertension or signs of end-organ damage. The American College of Obstetricians and Gynaecologists (ACOG) (14) suggest diagnosis in the presence of proteinuria and new-onset hypertension, or new-onset hypertension with thrombocytopenia, renal insufficiency, impaired liver function or pulmonary oedema. Classification of severe disease involves severe hypertension (systolic BP ≥ 160mmHg, diastolic BP ≥ 110mmHg or both) or signs of end-organ damage.

### Tregs in Pregnancy

Regulatory T-cells (Tregs) are a specialised subset of immunosuppressive cells defined by the expression of lineage-defining transcription factors FOXP3, CD25 and low or absent CD127 expression (15), subdivided into thymic/natural Tregs (nTregs) and peripherally induced Tregs (iTregs), which are thought to control autoimmune responses and mucosal immunity, respectively (16). Immunosuppressive function is primarily exerted by direct cell-cell interactions with the target cell, consumption of interleukin-2 (IL-2) and the release of anti-inflammatory molecules^14^. In addition to ensuring tolerance to self and non-inherited antigens (17), Tregs are essential in inducing transplantation tolerance (18–20).

During pregnancy, Tregs prevent rejection of the semi-allogeneic fetus by the maternal immune system (21, 22). Decidual Tregs, including nTregs and iTregs (23), create a tolerogenic microenvironment through the production of soluble factors such as IL-10 (22). In healthy pregnancies, great diversity in Treg populations exists in both peripheral blood and at the maternal-fetal interface (22). High levels of CD25^hi^FOXP3^+^ Tregs are found in decidual tissues^21^ and both FOXP3+ and FOXP3- Tregs are increased in the peripheral blood of pregnant women (22). The composition of fetal cells and maternal immune cells changes throughout gestation (22). T-cell frequencies increase during gestation, with local and systemic Treg expansion reaching its maximum in the 2^nd^ trimester (21). As labour progresses, the proportions of decidual Tregs once again decreases (24).

In PAO, maternal Tregs are altered (16). Treg maldistribution or functional impairment has been reported in implantation failure, miscarriage and pre-eclampsia (21, 22, 25, 26). In primary unexplained infertility, expression of FOXP3 mRNA is decreased in the uterine endometrium (27). In pre-eclampsia, Treg percentages are lower than in healthy pregnancies (28–30) and associated with spiral artery adaptation and defective maternal blood flow to the placenta (30). A reduction of decidual CD4+CD25^HI^ FOXP3+ and HELIOS+ Tregs is observed in miscarriages (21–23, 31).

Impaired peripheral Treg signalling has also been found (22). Signalling pathways implicated in modulating T-cell function during pregnancy include the IL-2–dependent STAT5ab signalling pathways (32), the PD1-PDL1 pathway (23) and the TIM-3 pathway (24). Fetal Tregs are also implicated (33), however, this systematic review and meta-analysis focuses on maternal Tregs only.

### Objective

Through this systematic review and meta-analysis, we aimed to determine whether there is an association between regulatory T cell levels and PAOs, including pre-eclampsia and preterm birth.

## 2 Materials and Methods

### Design

This study was conducted in accordance with the Preferred Reporting Items for Systematic Reviews and Meta-Analyses (PRISMA) guidelines (34). The review was prospectively registered with PROSPERO (CRD42020205469).

### Outcomes

Primary outcome was standardised mean difference (SMD) in Treg numbers between healthy pregnant women and women with pre-eclampsia or PTB. The measures used to identify these differences include Tregs expressing CD4+/CD25+/CD127low or CD4+/CD125+/FOXP3+ sampled from the peripheral blood or maternal decidua.

### Eligibility Criteria

Eligible for inclusion were original articles including clinical trials, case-control studies and cohort studies that examined the association between maternal Tregs in human pregnancy, sampled from the decidua or maternal blood, and the onset of pre-eclampsia and PTB. Studies were selected that compared these maternal co-morbidities with healthy age-matched pregnant individuals as control. No restrictions were made regarding population characteristics, such as age, ethnicity or setting. Studies examining fetal Tregs and studies that did not sample Tregs from the maternal blood or decidua were excluded. Duplicate studies were excluded from total counts.

### Information Sources and Search Strategy

Three reviewers (SG, KSR and MP) searched PubMed/MEDLINE, Embase and Cochrane CENTRAL for eligible articles published between August 1st, 2010 and August 1st, 2020 using search terms specific for ‘maternal’ OR ‘fetal’ ‘regulatory T-cells’ AND ‘pregnancy’, ‘pre-eclampsia’, ‘preterm birth’, OR ‘miscarriage’ (**Appendix 1**). Results were restricted by article type (see **Appendix 1** for detailed search strategies), language (English), and species (Human).

### Selection Process

For each article, title, abstract and full-text screening was performed independently by one of three reviewers (SG, KSR and MP). Screening results were reviewed by a senior author in the study (PS). Discrepancies were resolved through discussion in which a senior author was consulted (PS).

### Data Collection Process and Data Items

For each article, data was extracted independently by one of four reviewers (SG, KSR, MP, ASVC) using a predefined data extraction form. Results were reviewed by a senior author in the study (PS). From each study, information was extracted regarding study design, location, population, participant demographics, baseline characteristics, details of intervention and control, interventions and attrition rate. Miscellaneous information, e.g. method of delivery, antenatal steroid use was also recorded.

### Quality Assessment

A modified version (35) of the Newcastle–Ottawa Scale (NOS) (36) (**Supplementary Table 4**) was used to assess methodological quality of included studies. Studies were judged based on selection, comparability and outcome, with a maximum of 3, 2 and 2 stars, respectively, equating to a total score ranging from zero (worst) to 7 (best). ≥ 6 stars indicated high quality, 4–5 moderate quality and a high risk of bias and <4 indicated a very high risk of bias. Quality assessment was undertaken independently by two reviewers (KSR and MP), and inter-rater reliability was assessed.

### Statistical Analysis

We estimated the SMD in Treg numbers, sampled from the decidua and peripheral blood, of healthy pregnant women *versus* pregnant women with pre-eclampsia or PTB along with 95% confidence intervals (CI) under a random-effects (RE) model using Review Manager Version 5.4 (V.5.4.0) for Mac.

We used the tau-squared (Tau^2^), inconsistency index (I^2^), and chi-squared (χ²) test to quantify heterogeneity among different studies. Heterogeneity as defined by I^2^ was considered to be minor if 0% to 40%, moderate if 30% to 60%, substantial if 50% to 90% and considerable if 75% to 100%. The percent heterogeneity was interpreted in the context of the magnitude of the effect size and the strength of evidence surrounding the heterogeneity (37). Potential publication bias was tested using the rank correlation test of funnel plot asymmetry [Begg’s test (38) and Egger’s test (39)].

## Results

### Search Results

**Figure 1:**
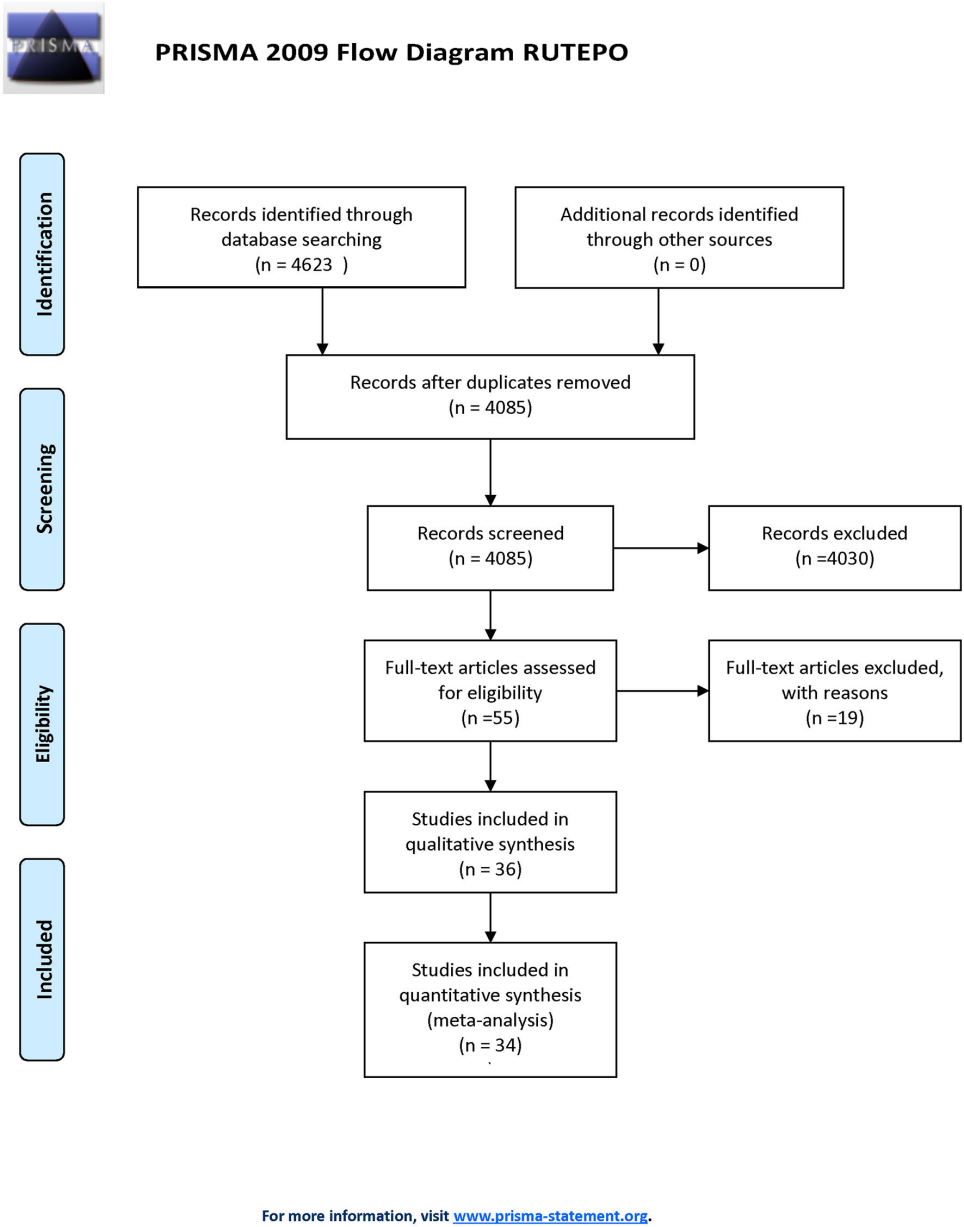
PRISMA flowchart of study selection.

**Figure 1** outlines the study selection process following PRISMA guidelines (40). The initial search identified 4,085 unique articles. 55 articles underwent full-text screening, with 36 studies included in qualitative synthesis and 34 in quantitative synthesis (meta-analysis). Treg populations between healthy pregnant women and pregnant women with pre-eclampsia was compared in 32 studies, 30 of which sampled Tregs from maternal peripheral blood (**Supplementray Table 1**). Four studies sample Tregs from maternal decidua (**Supplementary Table 2**), whilst an additional two studies compared Tregs, sampled from the peripheral blood, between healthy pregnant women and pregnant women who underwent PTL and PTB (**Supplementary Table 3**).

### Characteristics of Studies Included in the Meta-Analysis (Quantitative Synthesis)

**Supplementary Tables 1–3** feature the characteristics of included articles. Included studies were published between 2009 and 2019. Total sample sizes (including cases and controls) ranged from 20 to 108 pregnant subjects. Studies spanned 5 continents (Asia=19, Australia=1, Europe=9, North America=3 and South America=2), 12 countries (Australia=1, Bosnia and Herzegovina=1, Brazil=2, China=13, Czech Republic=1, Germany=1, Hungary=4, Iran=5, Japan=1, Mexico=1, Poland=2 and USA=2) and represented 1,783 participants (healthy controls=964, pre-eclampsia=759, PTB=60) of African American, Asian, Black, Caucasian, Hispanic, Latin and Persian ethnicity/race. The mean and median ages of women across studies ranged between 26.0 and 35.5 years. Treg analysis was typically performed in the second and third trimester of gestation. Most studies used flow cytometry (n=28) as the method of Treg analysis, while few reports using qPCR (n=5) and immunofluorescence tissue staining (n=1). Six studies considered and reported BMI measurements between cases and controls as an important confounder, and six studies considered and reported smoking status. All studies identified gestational age at the time of Treg analysis as a confounder, and 24 out of 34 studies (71%) considered gestational age at delivery. Birth weight was reported in 24 out of 34 studies (71%). A selection of different Treg markers were used in each study to identify Treg populations with CD4^+^, CD25^+^, FOXP3^+^ as well as CD4^+^, CD25^+^, CD127^low^ being the most common Treg marker combinations (**Supplementary Tables 1–3**). Gestational age at delivery is missing from 20 studies (**Supplementary Table 1**). Use of corticosteroids is not mentioned in 26 studies (**Supplementary Tables 1–3**).

### Meta-Analysis Findings (Quantitative Synthesis)

#### Lower Number of Tregs in Peripheral Blood in the Peripheral Blood of Women Who Develop Pre-Eclampsia

**Figure 2:**
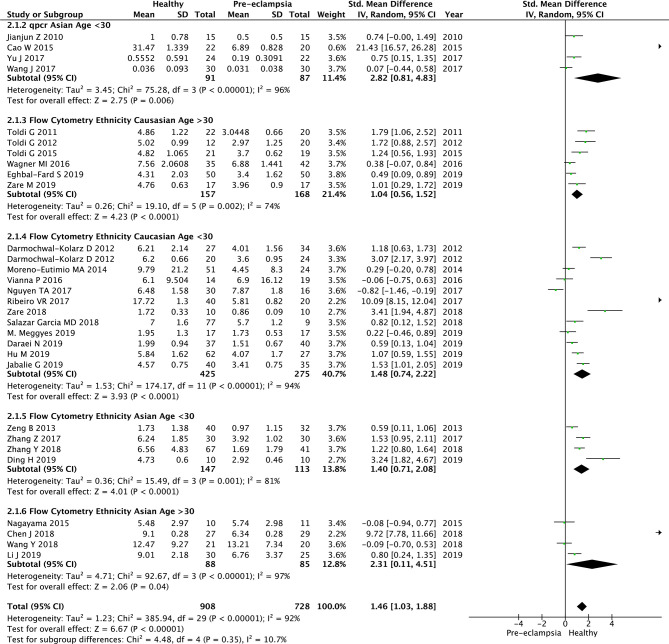
Standardized mean difference of T regulatory cell numbers in the peripheral blood of healthy pregnant women and women with pre-eclampsia, in subgroups according to ethnicity, age and method of analysis. CI, confidence interval; SD, standard deviation; Std. Mean Difference, standardised mean difference; IV, inverse variance.

30 studies were included in analysis exploring the association of pre-eclampsia and Treg populations in the peripheral blood. Twenty-six studies used flow cytometry to analyse Tregs populations (41–64) and four used qPCR (65–68). We analysed these two groups separately and combined them (**Figure 2**). In the qPCR group, the patients were matched for ethnicity and age group (Asian, <30 years old). The SMD of Treg numbers in the peripheral blood of healthy pregnant women compared to pregnant women with pre-eclampsia was 2.82 (95% CI, 0.81–4.83; I^2 =^ 96%; 4 studies), with healthy women reporting significantly higher Treg numbers in two studies and non-significant difference in two studies (**Figure 2.1.2**).

The overall SMD of Treg numbers in the peripheral blood of healthy pregnant women (using flow cytometry and qpcr) compared to pregnant women with pre-eclampsia was 1.46 (95% CI, 1.03–1.88; I^2 =^ 92%; 30 studies), with healthy women reporting significantly higher Treg numbers overall (**Figure 2**). We performed subgroup analysis based on their ethnic background and age. Testing for subgroup differences did not reveal any significant results (P=0.35, I^2 =^ 10.7%) (**Figure 2.1.2**, 2.1.3, 2.1.4, 2.1.5, 2.1.6). In addition changing the method of analysis from SMD to Mean Difference (MD) showed a MD of 2.49 (95% CI, 1.41-3.57; I^2 =^ 100%; 30 studies, data not shown). Testing for subgroup differences using MD did not show any significant results (P=0.14, I^2^-41.9%, data not shown). In addition, we also performed subgroup analysis based on the year of publication. We divided the studies to the ones published before (n=10) and after 2015 (n=20). Testing for subgroups differences based on the year of publication did not show any significant results (P=0.66, I^2 =^ 0%, data not shown).

#### Tregs in the Decidua of Women With Pre-Eclampsia and Healthy Women

**Figure 3:**
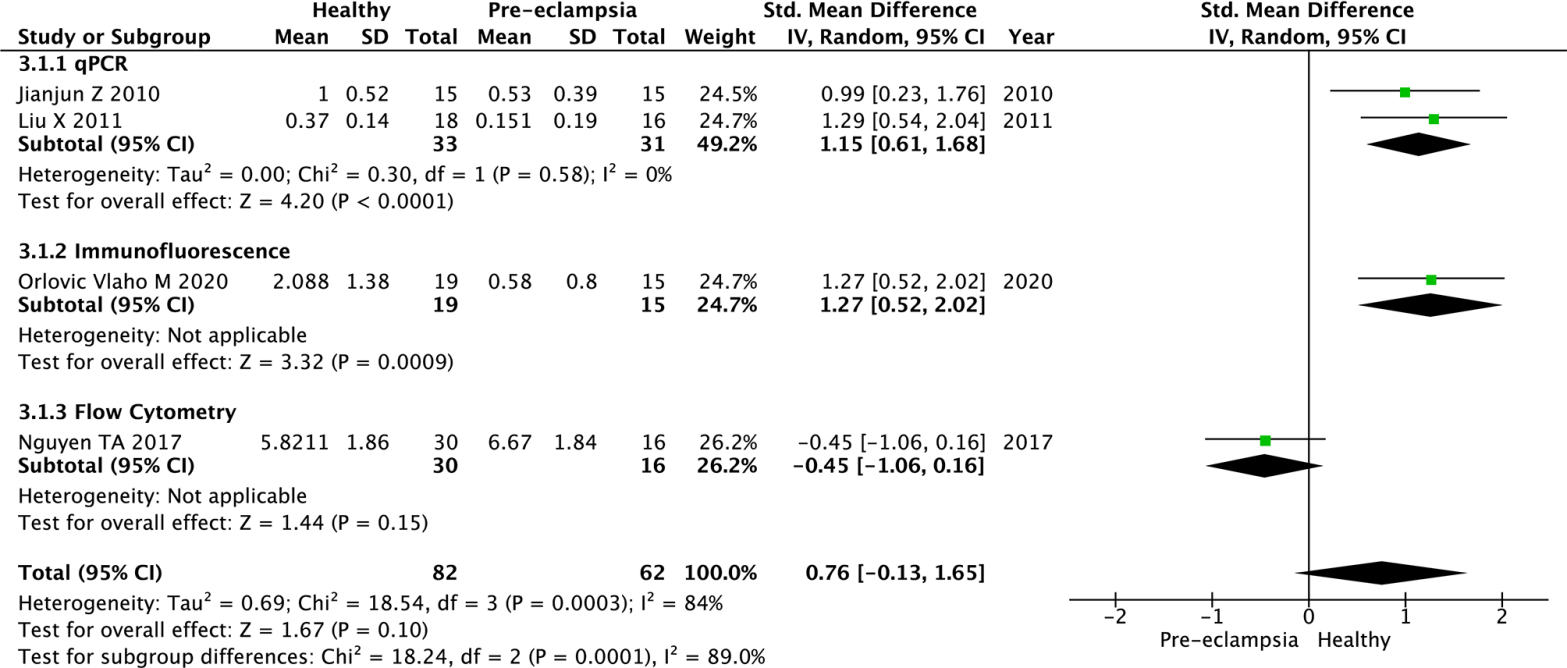
Standardized mean difference of T regulatory cell numbers in the decidua of healthy pregnant women and women with pre-eclampsia. CI, confidence interval; SD, standard deviation; Std. Mean Difference, standardised mean difference; IV, inverse variance.

Four studies were included in the analysis to determine association of pre-eclampsia and Treg populations in the maternal decidua. Two (69, 70) used qPCR to analyse Treg populations, one (71) used immunofluorescence tissue staining and one used flow cytometry (43). We analysed these three subgroups separately and further analysis combined all four studies (**Figure 3**). For the qPCR group (69, 70), the SMD in Treg numbers in the decidua of healthy pregnant women compared to pregnant women with pre-eclampsia was 1.15 (95% CI, 0.61–1.68; I^2 =^ 0%; 2 studies), with healthy pregnant women reporting significantly higher Treg numbers in both studies (**Figure 3.1.1**). The immunofluorescence tissue staining study (71) also reported significantly higher Tregs in healthy pregnant women (SMD, 1.27; 95% CI, 0.52–2.02) (**Figure 3.1.2**). Flow cytometry (43) showed no significant difference in Treg numbers between healthy pregnancies and women with pre-eclampsia (SMD, -0.45; 95% CI, -1.06–0.16) (**Figure 3.1.3**). Analysing qPCR, immunofluorescence tissue staining and flow cytometry studies together, the SMD of Treg numbers in the decidua of healthy pregnant women compared to pregnant women with pre-eclampsia was 0.76 (95% CI, -0.13–1.65; I^2^ = 84%; 4 studies), with healthy women reporting higher Treg numbers in 3 studies (65, 70, 71) and non-significant difference in 1 study (43). This result should be interpreted with caution as the 95% CI crosses the null value.

#### The Number of Tregs in Women With Early Pre-Eclampsia Are Similar to the Ones in Late Pre-Eclampsia

We identified studies, n=3 (44, 63, 68), which reported the number of Tregs separately in early *versus* late pre-eclampsia.

**Figure 4:**
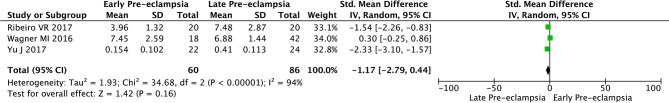
Standardized mean difference of T regulatory cell numbers in the maternal blood of women with early and late pre-eclampsia. CI, confidence interval; SD, standard deviation; Std. Mean Difference, standardised mean difference; IV, inverse variance.

The SMD in Treg numbers in the peripheral blood of pregnant women with late pre-eclampsia compared to pregnant women with early pre-eclampsia was -1.17 [95% CI, -2.79–0.44; I^2^ = 94%; 3 studies (44, 63, 68)], a non-significant difference with the 95% CI crossing the null value (**Figure 4**).

#### The Number of Tregs in Women Who Develop PTL and Had PTB Are Similar to Healthy Women

**Figure 5:**
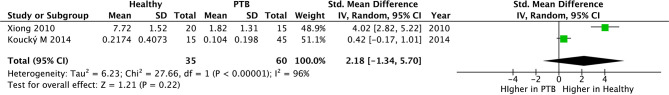
Standardized mean difference of T regulatory cell numbers in the peripheral blood of healthy pregnant women and women who underwent preterm birth (PTB). PTB, preterm birth; CI, confidence interval; SD, standard deviation; Std. Mean Difference, standardised mean difference; IV, inverse variance.

Only two studies (72, 73) reporting the association of PTB and Tregs in peripheral blood were included in the analysis. Both studies measured Tregs with flow cytometry. The SMD of Treg numbers in the peripheral blood of healthy pregnant women compared to pregnant women who underwent PTB was 2.18 (95% CI, -1.34–5.70; I^2 =^ 96%; 2 studies), with healthy pregnant women reporting higher Treg numbers overall in one study (72) and a non-significant difference in the second study (73) (**Figure 5**). However, the 95% CI crosses the null value.

### Heterogeneity of the Studies

Heterogeneity was considerable in all meta-analyses, the 95% prediction intervals for individual studies crossing the null value (**Figures 2–5**). I^2^ was 92% and 84% for the 30 and 4 respective studies investigating pre-eclampsia by sampling Tregs from maternal peripheral blood and decidua, respectively. Similarly, the I^2^ was 96% for the two studies investigating PTB by sampling Tregs from maternal peripheral blood. Where I^2^ could be estimated within subgroup analyses, age, ethnicity, year of publication and by the method of Treg analysis (flow cytometry *vs.* qPCR *vs.* immunofluorescence tissue staining), it remained considerable for all except the qPCR subgroup analysis of 2 studies (65, 70) investigating pre-eclampsia by sampling Tregs from the maternal decidua (I^2 =^ 0%) - 95% prediction intervals of neither of these studies crossed the null value (**Figure 3.1.1**). In the flow cytometry, ethnicity Caucasian, age >30 subgroup, of 6 studies (41, 52, 53, 56, 62, 63) the heterogeneity was relatively lower that the rest of the subgroups (I^2 =^ 74%) with only one study (63) crossing the null value. Heterogeneity should be considered as a confounder when interpreting the significance of results, particularly in relation to the analysis of pre-eclampsia studies sampling Tregs from the maternal decidua (**Figure 3**) and PTB studies sampling Tregs from the maternal peripheral blood (**Figure 5**) as the 95% CI crossed the null value in the end outcome of these analyses. This was not true for the analysis of pre-eclampsia studies sampling Tregs from the maternal peripheral blood (**Figure 2**).

### Publication Bias

**Figure 6:**
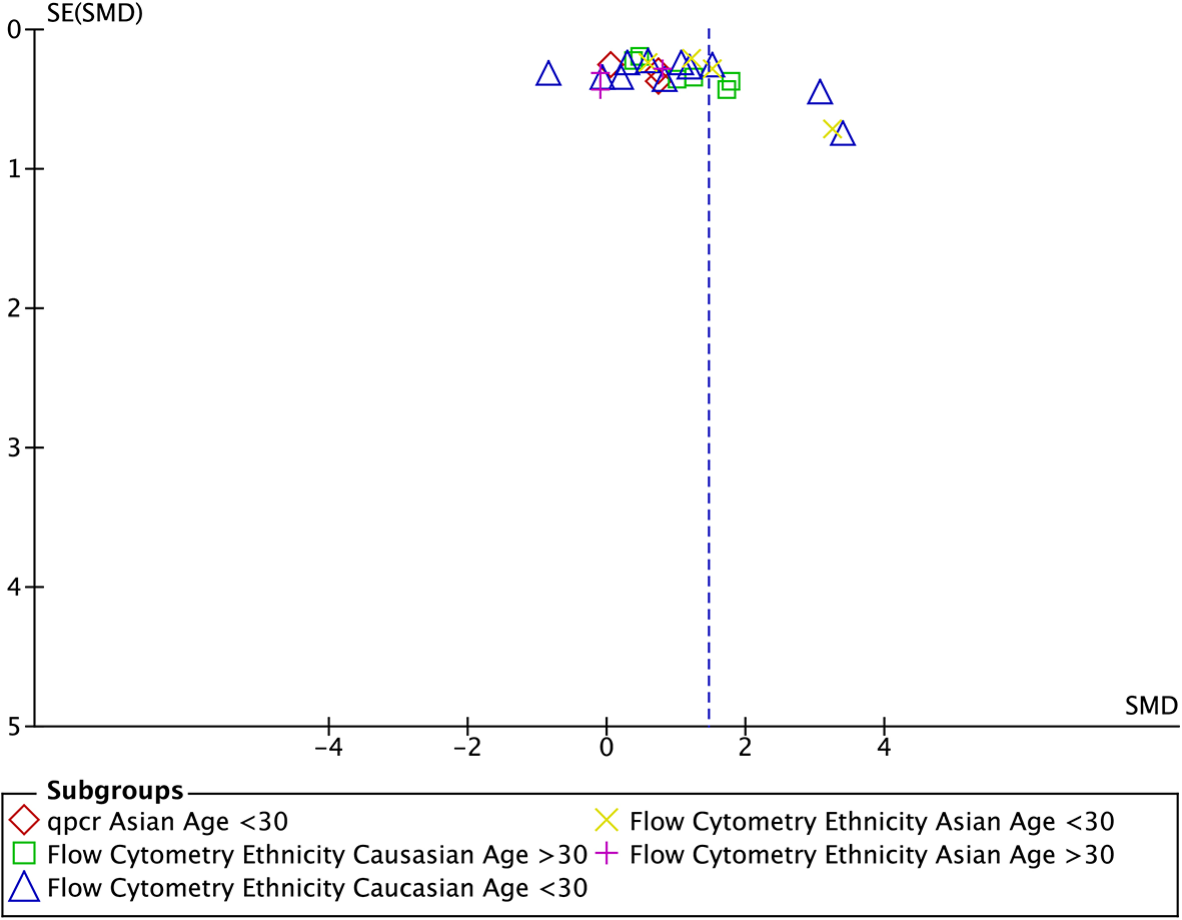
Funnel plot for studies looking at the number of Tregs in the maternal blood included in the subgroup meta-analysis (n=30). SE, standard error; SMD, standardised mean difference.

A funnel plot was used to graphically evaluate articles for publication bias. This was tested using the rank correlation test of funnel plot asymmetry [Begg’s test (38) and Egger’s test (39)]. SMD values were plotted against standard error (SE). Data from the 30 studies seemed to be roughly symmetrically distributed in an inverted funnel-shaped area (**Figure 6**).

### Quality Assessment

The overall quality rating of the studies included in the meta-analysis was moderate, representing a high risk of bias. NOS scores ranged between 3–6 with a median score of 4 out of 7 (**Supplementray Table 5**). Quality ratings were impaired by poor population representativeness of the exposed cohort (due to hospital-based sampling), and inadequate follow up (<1 month after labour). Comparability was limited in several studies that did not adjust for all confounding variables (age, race, smoking and interpregnancy interval). The IRR was 100% regarding the assessment of ‘selection’ and ‘outcomes’ criteria; however, there were inter-rater discrepancies in the assessment of ‘comparability’ in 13 out of 36 studies (IRR=64%).

## Discussion

### Findings

Our meta-analysis suggests that lower Treg cell numbers may be a potential independent risk factor for PAO, including pre-eclampsia and potentially PTL. Overall, healthy pregnant women have significantly higher Treg numbers than pregnant women with pre-eclampsia, evident by an SMD of 1.46 (95% CI, 1.03–1.88; I^2 =^ 92%; 30 studies) when sampling Tregs from the peripheral blood. In studies sampling Tregs from the maternal decidua, healthy pregnant women had higher, but non-significantly higher Treg numbers compared to women with pre-eclampsia, evident by an SMD of 0.76 (95% CI, -0.13–1.65; I^2^ = 84%; 4 studies). This might be due to poor phenotyping of the decidual tissue. Healthy pregnant women also have a non-significant numerically higher Treg numbers compared to pregnant women who undergo PTL, with an SMD of 2.18 (95% CI, -1.34–5.70; I^2 ^= 96%; 2 studies) when sampling Tregs from the peripheral blood. Similar trends are observed in subcohort analysis when studies are grouped by the method of Treg analysis.

This is supported by previous research by Han et al (74) and Schober et al (75) who found an association between impaired Treg function and pre-eclampsia (74) and PTL (75). Han et al. (74), using high-dimensional mass cytometry immunoassay, suggests that specific aspects of peripheral immune system dynamics may be disrupted in preeclamptic pregnancies. Furthermore, Schober et al. (75), using flow cytometry, found that the suppressive activity of CD4+CD127low+/-CD25+-Treg cells was strongly diminished in PTL women and, to a lesser extent, in spontaneously term labouring women compared to term non-labouring women. This reduction in suppressive activity was due to Treg-cell deficiency but not due to CD4+-responder T (Tresp) cell resistance, with CD4+-T cells significantly reduced in term and preterm labouring women (75). There is a need for additional research, using tightly phenotyped PTL groups, before confirming any relationship and methods used by Han et al. and Schober et al. may be worthwhile.

### Strengths

We identified and screened over 4,000 unique articles, the meta-analysis therefore including participants across five continents of different ethnicity, adding to the representability and generalizability of findings. We found similar results, in terms of Treg associations with healthy and adverse pregnancy outcomes, across studies that sampled Tregs from different sites (maternal decidua and maternal peripheral blood) as well as studies using other methods of Treg analysis (flow cytometry, qPCR and immunofluorescence tissue staining), increasing robustness of findings. Performance of subcohort analysis by age, ethnicity, year of publication, site of sampling and method of Treg analysis further supports the strength of association across different research conditions. Most studies attempted to adjust for confounding variables.

### Limitations

Participant numbers were limited, especially for the PTB cohort (1739 pregnant women including 944 healthy controls, 735 pre-eclampsia, 60 PTB). Furthermore, each separate study had a small number of patients in each group. Indeed, only two studies investigating PTB were included in quantitative analysis; both sampling Tregs from maternal peripheral blood, none sampling decidua. In pre-eclampsia, only four studies tested Tregs from the maternal decidua, utilising three different methods of Treg analysis, with the flow cytometry study producing opposing findings to studies utilising qPCR or immunofluorescence tissue staining. Antenatal corticosteroids have been shown to alter T cell trafficking and cytokine production (76), yet use of corticosteroids prior to blood sampling was omitted in most studies (supplementary data). Additionally, whilst Treg cells are classified into naïve and effector Tregs, which express weak and powerful immunoregulation respectively, we did not discriminate between the two. Comparing Treg populations in early onset *versus* late onset pre-eclampsia is also important given the distinct underlying pathogenic processes (44, 63, 68, 77). In our cohort only 3 studies reported separately the Treg numbers in early *versus* late pre-eclampsia. In addition a subgroup analysis based on BMI would have been ideal since there is evidence the BMI alters the number of Tregs in obese individuals (78). Unfortunately only 7 studies reported BMI in their results, which was between 22-28 and no subgroup analysis could have been done (**Supplementary Tables 1–3**).

Methodological quality of studies was low to moderate. Nevertheless, all studies were included in the analysis regardless of quality assessment, none excluded based on high risk of bias. It may have been prudent to repeat the analysis, excluding studies with low NOS quality rating. Equally, meta-analyses could have been repeated after excluding studies with high heterogeneity (I^2^) in which the 95% prediction intervals crossed the null value (Figs. 2-5). Overall, there was considerable heterogeneity across analyses. Indeed, this limited the significance of results in three of the meta-analyses, specifically for pre-eclampsia studies sampling Tregs from the maternal decidua and PTB studies sampling Tregs from the maternal peripheral blood as well as in the early *versus* late comparison (**Figure 4**). Assessing relative risk (RR) or odds ratio (OR) in addition to SMD may have demonstrated a stronger level of association to support outcomes.

### Clinical Significance

Future research could investigate the potential of monitoring Treg numbers in peripheral blood of pregnant women as a possible biomarker to assess the risk of PAO, stratifying patients with high-risk pregnancies. Future preclinical and clinical models could investigate strategies to increase Treg numbers in pregnant women as a candidate therapeutic approach. Both of these suggestions, however, remain at a hypothesis stage and require further systematic evaluation.

## Conclusion

This meta-analysis suggests an association between lower T-regulatory cell numbers and risk for pre-eclampsia and potentially for PTL (28, 29). Importantly, correlation does not imply causation and possibility of an underlying mechanism causing both low Treg numbers and pre-eclampsia and PTL must be considered. As the precise mechanisms by which Tregs may mediate pre-eclampsia and PTL remain unclear, further research is necessary to elucidate the underlying processes and highlight the causative link.

## Data Availability Statement

The original contributions presented in the study are included in the article/**Supplementary Material**. Further inquiries can be directed to the corresponding author.

## Author Contributions

PS, SG, MP, KR, and AS conceptualized the topic and structure of the systematic review. SG, MP, KR, and AS drafted and revised the manuscript. AE, NM, KD, CS, GL, RT, KN, and PS provided expert opinion, edited, and approved the final manuscript. All authors contributed to the article and approved the submitted version.

## Funding

PS is funded by an NIHR Clinical Lectureship (CL-2018-17-002). This study was funded by the Fetal Medicine Foundation (KHN,AE&NM) (registered charity 1037116), Tommy’s (RT&KD) (registered charity number 1060508) and the National Institute for Health Research (NIHR) Biomedical Research Centre at Guy’s and St Thomas’ National Health Service Foundation Trust and King’s College London (IS-BRC-1215–20006).

## Author Disclaimer

The views expressed in this Article are those of the authors and not necessarily those of the National Health Service, the NIHR, or the Department of Health.

## Conflict of Interest

The authors declare that the research was conducted in the absence of any commercial or financial relationships that could be construed as a potential conflict of interest.

## Publisher’s Note

All claims expressed in this article are solely those of the authors and do not necessarily represent those of their affiliated organizations, or those of the publisher, the editors and the reviewers. Any product that may be evaluated in this article, or claim that may be made by its manufacturer, is not guaranteed or endorsed by the publisher.

## Appendix 1 Search Terms

### 1. PubMed

**Theme 1: Maternal T Cells in Pregnancy**

- ((maternal) OR (mother)) AND ((regulatory T cells) OR (Tregs)) AND (pregnancy)

**Theme 2: Fetal T Cells in Pregnancy**

- ((Fetal) OR (Fetal)) AND ((Regulatory T Cells) OR (Tregs) OR (Regulatory T Lymphocyte)) AND (Pregnancy)

**Theme 3: Adverse Outcomes**

- ((Preeclampsia) OR (preterm birth) OR (miscarriage)) AND ((Regulatory T cells) OR (Tregs) OR (Regulatory T lymphocyte)) AND (pregnancy)

### 2. Embase

**Theme 1: Maternal T Cells in Pregnancy**

- ((maternal OR mother) AND ‘regulatory t lymphocytes’ OR ‘regulatory t cells’ OR ‘tregs’/exp OR tregs) AND (‘pregnancy’/exp OR pregnancy)

**Theme 2: Fetal T Cells in Pregnancy**

- ((((Fetal OR Fetal) AND ‘Regulatory T Cells’ OR Tregs OR Regulatory T Lymphocyte) AND Pregnancy))

**Theme 3: Adverse Outcomes**

- ((preeclampsia) OR ‘preterm birth’ OR ‘preterm labor’ AND ‘tregs’) AND (‘pregnancy’)

### 3. Cochrane

**Theme 1: Maternal T Cells in Pregnancy**

- (maternal):ti,ab,kw OR (mother):ti,ab,kw AND (Tregs):ti,ab,kw OR (T regulatory lymphocytes):ti,ab,kw OR (T regulatory cells):ti,ab,kw AND (pregnancy):ti,ab,kw

**Theme 2: Fetal T Cells in Pregnancy**

- (Fetal):ti,ab,kw OR (Fetal):ti,ab,kw AND (Tregs):ti,ab,kw OR (Regulatory T Cells):ti,ab,kw OR (Regulatory T Lymphocytes):ti,ab,kw AND (Pregnancy):ti,ab,kw

**Theme 3: Adverse Outcomes**

- (preeclampsia):ti,ab,kw OR (preterm birth):ti,ab,kw OR (miscarriage):ti,ab,kw AND (Tregs):ti,ab,kw OR (regulatory T lymphocyte):ti,ab,kw OR (regulatory T cells):ti,ab,kw AND (pregnancy):ti,ab,kw
