# Supplementary material for: Regulatory T Cells in Pregnancy Adverse Outcomes: A Systematic Review and Meta-Analysis
Source: Front Immunol. 2021 Oct 29;12:737862. doi: 10.3389/fimmu.2021.737862 (PMC8586555; doi:10.3389/fimmu.2021.737862)
Supplement: Supplementary file 1 [file DataSheet_1.docx]

***Supplementary Table 1***

| **Author** | **Year** | **Location** | **Study Design** | **Final N PET /Healthy PBMCs** | **Ethnicity /Race** | **Age group (years)** | **BMI** | **Smoking (n)** | **Blood pressure systolic (mmHg)** | **Blood pressure diastolic (mmHg)** | **Gestation at delivery (weeks)** | **Gestation at Treg analysis (weeks)** | **Steroids prior to isolation** | **Birth weight (grams)** | **Tregs marker** | **Method of analysis** |
| --- | --- | --- | --- | --- | --- | --- | --- | --- | --- | --- | --- | --- | --- | --- | --- | --- |
| Jianjun Z* | 2010 | China | Comparative Study | 15 PET | Asian | 27.2 ± 2.3 | - | - | 152.8 ± 12.8 | 100.4 ± 9.4 | 35 ± 2.6 | 35 ± 2.6 | Yes | 2797 ± 759.4 | FOXP3 mRNA | qPCR |
|  |  |  |  | 15 Healthy | Asian | 28.6 ± 2.5 | - | - | 114 ± 10 | 74 ± 12.9 | 37.9 ± 1.1 | 37.9 ± 1.1 | Yes | 3345 ± 336.6 | FOXP3 mRNA | qPCR |
| Toldi G | 2011 | Hungary | Comparative Study | 20 PET | Caucasian | 32.3±7.58 | - | - | 163.2±24.73 | 103±8.78 | 35.56±6.38 | 34.56±6.38 | No information | 2524.6±1663.51 | CD25+ FOXP3+ | Flow cytometry |
|  |  |  |  | 22 Healthy | Caucasian | 33.1±4.75 | - | - | 110±6.34 | 65.85±7.92 | 39±1.58 | 35.64±2.37 | No information | 3293.3±491.34 | CD25+ FOXP3+ | Flow cytometry |
| Darmochwal-Kolarz D | 2012 | Poland | prospective observational cohort study | 34 PET | Caucasian | 28 ± 4.76 | - | - | 155.35 ± 15.85 | 96.47 ± 5.66 | - | 34.05 ± 2.14 | No | 2560 ± 615 | CD4+ CD25+ FOXP3+ | Flow cytometry |
|  |  |  |  | 27 Healthy | Caucasian | 27.68 ± 5.31 | - | - | 110.25 ± 20.15 | 73.56 ± 7.18 | - | 36.62 ± 1.63 | No | 3280 ± 365 | CD4+ CD25+ FOXP3+ | Flow cytometry |
| Toldi G | 2012 | Hungary | Comparative Study | 20 PET | Caucasian | 32.3±3.5 | 27.69±6.46 | 2 | 150±15.96 | 97.48±5.59 | 34.86±4.79 | 33.86±4.79 | No information | 2037.3±1148.9 | CD4+ CD25+ FOXP3+ | Flow cytometry |
|  |  |  |  | 20 Healthy | Caucasian | 32.9±5.59 | 23.51±5.66 | 0 | 113.6±7.98 | 68.92±13.56 | 38.36±0.79 | 37±1.59 | No information | 3403.3±223.4 | CD4+ CD25+ FOXP3+ | Flow cytometry |
| Darmochwal-Kolarz D | 2012 | Poland | Comparative Study | 24 PET | Caucasian | 28.68 ± 4.76 | - | - | 155.35 ± 15.85 | 96.47 ± 5.66 | - | 34.05 ± 2.14 | No | 2560 ± 615 | CD4+CD25+FOXP3+ | Flow cytometry |
|  |  |  |  | 20 Healthy | Caucasian | 27.68 ± 5.31 | - | - | 110.25 ± 20.15 | 73.56 ± 7.18 | - | 34.62 ± 1.63 | No | 3280 ± 365 | CD4+CD25+FOXP3+ | Flow cytometry |
| Zeng B | 2013 | China | prospective observational cohort study | 32 PET | Asian | 29.5 ± 5.1 | - | - | 157 ± 14.4 | 105.4 ± 11.1 | 36.6 ± 2.1 | 36.6 ± 2.1 | No information | - | CD4+ CD25+ FOXP3+ | Flow cytometry |
|  |  |  |  | 40 Healthy | Asian | 29.5 ± 4.3 | - | - | 116.7 ± 10.3 | 77.2 ± 9.8 | 37.2 ± 1.1 | 37.2 ± 1.1 | No information | - | CD4+ CD25+ FOXP3+ | Flow cytometry |
| Moreno-Eutimio MA | 2014 | Mexico | case-control study | 24 PET | Latin/Hispanic | 23.78 ± 5.68 | - | - | 175.3 ± 11.28 | 119.3 ± 6.22 | 35.23±7.88 | 35.23±7.88 | No information | 1737.59±2088.35 | CD4+ CD25high FOXP3+ | Flow cytometry |
|  |  |  |  | 51 Healthy | Latin/Hispanic | 26.04 ± 7.37 | - | - | 110.3 ± 18.15 | 71.5 ± 6.32 | 38.41±4.58 | 38.41±4.58 | No information | 2482.52±2341 | CD4+ CD25high FOXP3+ | Flow cytometry |
| Cao W | 2015 | China | prospective observational cohort study | 20 PET | Asian | 26.37 ± 4.1 | - | 1 | 167.25 ± 8.07 | 116.33 ± 8.15 | - | 38.57 ± 0.98 | No | - | FOXP3 mRNA | qPCR |
|  |  |  |  | 22 Healthy | Asian | 27.38 ± 3.38 | - | 1 | 110.25 ± 8.07 | 75.11 ± 5.58 | - | 36.62 ± 1.57 | No | - | FOXP3 mRNA | qPCR |
| Nagayama S | 2015 | Japan | case-control study | 11 PET | Asian | 35.5 ± 3.5 | - | 1 | 165 ± 8 | 99 ±8 | 34.8 ± 4.0 | 33.9 ± 4.0 | No information | 2024 ±795 | CD4+ CD25+ FOXP3+ | Flow cytometry |
|  |  |  |  | 10 Healthy | Asian | 32.8 ± 5.5 | - | 0 | 143 ± 35 | 87 ± 19 | 36.8 ± 2.5 | 29.7 ± 4.2 | No information | 2336 ± 704 | CD4+ CD25+ FOXP3+ | Flow cytometry |
| Toldi G | 2015 | Hungary | Comparative Study | 19 PET | Caucasian | 31±4.80 | - | - | 160±32.04 | 100.4±28.03 | 34.64±5.61 | 34±4.81 | No information | 3773.6±1081.31 | CD4+ CD25high FOXP3+/CD4+ | Flow cytometry |
|  |  |  |  | 21 Healthy | Caucasian | 30±3.97 | - | - | 113.2±19.88 | 68.2±19.87 | 39±3.18 | 36±1.59 | No information | 3282.51±620.12 | CD4+ CD25hi FOXP3+/CD4+ | Flow cytometry |
| Wagner MI | 2016 | Germany | Comparative Study | 42 PET | Caucasian | 33±18.42 | - | - | - | - | 32.87±13.82 | 32.87±13.82 | No information | - | CD4+ CD127low FOXP3+ | Flow cytometry |
|  |  |  |  | 41 Healthy | Caucasian | 32±14.59 | - | - | - | - | 40±3.07 | 40±3.07 | No information | - | CD4+ CD127low FOXP3+ | Flow cytometry |
| Vianna P | 2016 | Brazil | case-control study | 19 PET | Caucasian | 28.58 ± 1.22 | - | 1.96 ± 0.9 | - | - | - | 31.9 ± 1.48 | No information | - | CD4+ CD25bright FOXP3+ | Flow cytometry |
|  |  |  |  | 14 Healthy | Caucasian | 26.16 ± 1.01 | - | 1.00 ± 0.5 | - | - | - | 30.9 ± 1.09 | No information | - | CD4+ CD25bright FOXP3+ | Flow cytometry |
| Wang J | 2017 | China | case-control study | 30 PET | Asian | 27.8 ± 2.5 | - | - | - | - | - | 34.1 ± 2.3 | No information | - | FOXP3 mRNA | qPCR |
|  |  |  |  | 30 Healthy | Asian | 27.7 ± 3.1 | - | - | - | - | - | 33.7 ± 1.1 | No information | - | FOXP3 mRNA | qPCR |
| Nguyen TA* | 2017 | USA | prospective observational cohort study | 16 PET | Caucasian, Hispanic, Black, Asian | 30.8 ± 2.6 | - | 0 | - | - | 38.6 ± 0.6 | 38.6 ± 0.6 | No information | 3231 ± 191 | FOXP3+ CD4+ | Flow cytometry |
|  |  |  |  | 30 Healthy | Caucasian, Hispanic, Black, Asian | 28.8 ± 2.1 | - | 1 | - | - | 39.1 ± 0.2 | 39.1 ± 0.2 | No information | 3231 ± 191 | FOXP3+ CD4+ | Flow cytometry |
| Ribeiro VR | 2017 | Brazil | case-control study | 20 PET | Caucasian | 27±20.74 | - | - | 158.6±31.91 | 103.6±23.93 | 37±4.49 | 37±4.49 | No information | - | CD4+ CD25+ FOXP3+ | Flow cytometry |
|  |  |  |  | 10 Healthy | Caucasian | 27.48±15.48 | - | - | 101±12.90 | 63.69±8.6 | 37±5.16 | 37±5.16 | No information | - | CD4+ CD25+ FOXP3+ | Flow cytometry |
| Yu J | 2017 | China | Case-control | 22 PET | Asian | 26.7 ± 4.3 | - | - | 166.9 ± 8.0 | 116.6 ± 8.2 | 35.3 ± 1.2 | 35.3 ± 1.2 | No | 2976.8 ± 232.8 | FOXP3 mRNA | qPCR |
|  |  |  |  | 24 Healthy | Asian | 26.0 ± 4.4 | - | - | 111.0 ± 9.8 | 72.5 ± 8.0 | 36.5 ± 1.0 | 36.5 ± 1.0 | No | 3421.8 ± 205.9 | FOXP3 mRNA | qPCR |
| Zhang Z | 2017 | China | case-control study | 30 PET | Asian | 29.79 ± 5.40 | - | - | 169.57 ± 20.72 | 112.11 ± 17.17 | 36.01 ± 1.12 | 33.00 ± 2.95 | No | 2023.21 ± 728.71 | CD4+ CD25+ CD127low | Flow cytometry |
|  |  |  |  | 30 Healthy | Asian | 30.33 ± 3.99 | - | - | 113.93 ± 9.82 | 72.30 ± 7.00 | 39.00 ± 0.67 | 33.00 ± 3.87 | No | 3272.22 ± 507.32 | CD4+ CD25+ CD127low | Flow cytometry |
| Zare M | 2018 | Iran | case-control study | 10 PET | Persian | 29.7 ± 1.9 | - | - | 134 ± 6.9 | 93 ± 4.8 | - | 34.4 ± 2 | No information | - | CD4+ CD25+ FOXP3+ | Flow cytometry |
|  |  |  |  | 10 Healthy | Persian | 28.6 ± 2.0 | - | - | 104 ± 5.1 | 73±4.8 | - | 33.6 ± 2 | No information | - | CD4+ CD25+ FOXP3+ | Flow cytometry |
| Wang Y | 2018 | China | case-control study | 20 PET | Asian | 33.04±11.97 | - | - | 164.84±50.26 | 121.64±23.14 | 32.3±10.37 | 32.3±10.37 | Yes to three patients | 2507.61±1954 | CD4+ CD25+ CD127low | Flow cytometry |
|  |  |  |  | 21 Healthy | Asian | 27.95±11.92 | - | - | 124.22±23.06 | 80±18.29 | 38.16±3.18 | 38.16±3.18 | No | 3560±1009.7 | CD4+ CD25+ CD127low | Flow cytometry |
| Salazar Garcia MD | 2018 | USA | prospective cohort study | 9 PET | African American 88.9%, Hispanic 11.1% | 24.0 ± 4.3 | - | 5 | 164.4 ± 29.0 | 120.3 ± 13.0 | 36.7 ± 1.7 | 11.6 ± 2.7 | No information | 2552.2 ± 610.4 | CD4+ CD25bright CD127dim/− | Flow cytometry |
|  |  |  |  | 77 Healthy | African American 38.9%, Hispanic 54.6% | 26.6 ± 5.6 | - | 0 | 101.7 ± 15.1 | 72.3 ± 9.1 | 38.5 ± 2.2 | 10.4 ± 2.6 | No information | 3173.4 ± 601.5 | CD4+ CD25bright CD127dim/− | Flow cytometry |
| Chen J | 2018 | China | case-control study | 29 PET | Asian | 31 ± 7 | 28.7 ± 6.3 | - | 155 ± 49 | 100 ± 36 | 37 ± 2.1 | 28.7 ± 6.3 | No information | 2.932 ± 378 | CD4+ CD25+ CD127- | Flow cytometry |
|  |  |  |  | 27 Healthy | Asian | 26 ± 6 | 26.0 ± 5.9 | - | 119 ± 30 | 77 ± 26 | 39 ± 2.2 | 26.0 ± 5.9 | No information | 3.212 ± 212 | CD4+ CD25+ CD127- | Flow cytometry |
| Zhang Y | 2018 | China | Prospective, case–control, comparative study | 41 PET | Asian | 29.70 ±4 .27 | - | - | 144.40 ± 10.18 | 94.00 ± 8.59 | - | 37.13 ± 1.26 | No information | - | CD4+ CD25high CD127low | Flow cytometry |
|  |  |  |  | 67 Healthy | Asian | 27.60 ± 3.76 | - | - | 119.50 ± 9.93 | 74.67 ± 7.87 | - | 36.92 ± 1.24 | No information | - | CD4+ CD25high CD127low | Flow cytometry |
| Daraei N | 2019 | Iran | case-control study | 40 PET | Persian | 28.5 ± 0.925 | - | - | 160 ± 3.325 | 90 ± 3.021 | - | 32 ± 0.534 | No information | - | CD4+ CD25high | Flow cytometry |
|  |  |  |  | 37 Healthy | Persian | 26.5 ± 0.821 | - | - | 117.5 ± 1.212 | 70 ± 1.341 | - | 34 ± 0.322 | No information | - | CD4+ CD25high | Flow cytometry |
| Ding H | 2019 | China | case-control study | 10 PET | Asian | 29.90 ± 1.40 | 27.90 ± 1.06 | - | 159.40 ± 11.28 | 104.80 ± 7.09 | - | 31.64 ± 0.87 | No information | - | CD4+ CD25+ FOXP3+ | Flow cytometry |
|  |  |  |  | 10 Healthy | Asian | 29.80 ± 1.65 | 26.27 ± 1.36 | - | 117.90 ± 2.42 | 75.20 ± 2.63 | - | 32.07 ± 0.64 | No information | - | CD4+ CD25+ FOXP3+ | Flow cytometry |
| Eghbal-Fard S | 2019 | Iran | case-control study | 50 PET | Persian | 33.2 ± 5.1 | 28.4 ± 2.9 | - | 156.1 ± 14.9 | 100.2 ± 20.1 | 35.1 ± 4.8 | 35.8 ± 5.0 | No | 2770.1 ± 880.8 | CD4+ CD25+ CD127− | Flow cytometry |
|  |  |  |  | 50 Healthy | Persian | 31.8 ± 3.4 | 27.5 ± 3.7 | - | 115.5 ± 15.1 | 70.1 ± 15.8 | 38.9 ± 2.7 | 37.1 ± 2.3 | No | 3335.3 ± 320.1 | CD4+ CD25+ CD127− | Flow cytometry |
| Hu M | 2019 | Australia | Prospective, case–control, comparative study | 27 PET | Caucasian | 28.9 ± 6 | - | - | - | - | - | 35.4 ± 3.2 | No information | 2527 ± 1054 | CD4+ CD25+ FOXP3+ | Flow cytometry |
|  |  |  |  | 62 Healthy | Caucasian | 30.3 ± 4.8 | - | - | - | - | - | 39.2 ± 0.9 | No information | 3558 ± 451 | CD4+ CD25+ FOXP3+ | Flow cytometry |
| Jabalie G | 2019 | Iran | Comparative Study | 35 PET | Persian | 29.47 ± 4.50 | 23.52 ± 3.93 | - | 144.49 ± 8.12 | 90.88 ± 10.21 | 37.1 ± 2.80 | 34.21 ± 1.22 | No information | 2865.1 ± 771.2 | CD4+ CD25+ CD127− | Flow cytometry |
|  |  |  |  | 40 Healthy | Persian | 28.30 ± 3.50 | 22.12 ± 3.72 | - | 114.5 ± 15.1 | 70.2 ± 13.8 | 39.1 ± 1.90 | 34.17 ± 1.10 | No information | 3232.5 ± 301.1 | CD4+ CD25+ CD127− | Flow cytometry |
| Li J | 2019 | China | patient control study | 25 PET | Asian | 32.0 ± 4.6 | 28.2 ± 2.9 | - | 159 ± 13 | 94 ± 13 | 36.64±2.35 | 36.64±2.35 | Yes | 2715 ± 1015 | CD4+ CD25+ FOXP3+ | Flow cytometry |
|  |  |  |  | 30 Healthy | Asian | 30.0 ± 2.9 | 27.0 ± 2.1 | - | 121 ± 8 | 70 ± 10 | 38.64±0.77 | 36.65±2.34 | No | 3523 ± 338 | CD4+ CD25+ FOXP3+ | Flow cytometry |
| Zare M | 2019 | Iran | case-control study | 17 PET | Persian | 31.2 ± 2.4 | - | - | 141.0 ± 8.8 | 91.5 ± 5.7 | 35.3 ± 3.6 | 33.9 ± 3.5 | No information | 2240 ± 775 | CD4+ CD25high CD127low | Flow cytometry |
|  |  |  |  | 17 Healthy | Persian | 29.6 ± 2.7 | - | - | 111.6 ± 8.8 | 71.6 ± 6.0 | 39.0 ± 0.7 | 35.1 ± 03.7 | No information | 2992.5 ± 771 | CD4+ CD25high CD127low | Flow cytometry |
| Meggyes M | 2019 | Hungary | case-control study | 17 PET | Caucasian | 29.00±20.21 | - | - | - | - | 32.35 ± 3.46 | 30.76 ± 2.41 | No information | 1631.76 ± 738.56 | CD4+ CD25+ FOXP3+ | Flow cytometry |
|  |  |  |  | 17 Healthy | Caucasian | 33.9±13.74 | - | - | - | - | 39.14 ± 1.10 | 33.29 ± 3.96 | No information | 3444.29 ± 512.38 | CD4+ CD25+ FOXP3+ | Flow cytometry |

***Study characteristics for meta-analysis comparing T regulatory cell numbers in the peripheral blood of healthy pregnant women and women with pre-eclampsia toxaemia (PET); n=30.***

*PBMCs peripheral blood mononuclear cells.*

**Studies reporting both peripheral blood and decidual Treg analysis.*

***Supplementary Table 2***

| **Author** | **Year** | **Location** | **Study Design** | **Final N PET /Healthy Decidua** | **Ethnicity/Race** | **Age group (years)** | **BMI** | **Smoking (%, n)** | **Blood pressure systolic (mmHg)** | **Blood pressure diastolic (mmHg)** | **Gestation at delivery (weeks)** | **Gestation at Treg analysis (weeks)** | **Steroids prior to isolation** | **Birth weight (grams)** | **Tregs marker** | **Method of analysis** |
| --- | --- | --- | --- | --- | --- | --- | --- | --- | --- | --- | --- | --- | --- | --- | --- | --- |
| Jianjun Z* | 2010 | China | Comparative Study | 15 PET | Asian | 27.2 ± 2.3 | - | - | 152.8 ± 12.8 | 100.4 ± 9.4 | 35.0 ± 2.6 | 35.0 ± 2.6 | Yes | 2797.3 ± 759.4 | FOXP3 | qPCR |
|  |  |  |  | 15 Healthy | Asian | 28.6 ± 2.5 | - | - | 114 ± 10.0 | 74.0 ± 12.9 | 37.9 ± 1.1 | 37.9 ± 1.1 | Yes | 3345 ± 336.6 | FOXP3 | qPCR |
| Liu X | 2011 | China | prospective observational cohort study | 16 PET | Asian | 31.8 ± 4.5 | - | - | 179.8 ± 28.0 | 116.2 ± 18.1 | 35.7 ± 2.0 | 35.7 ± 2.0 | No Information | 2447.5 ± 689.7 | FOXP3 | qPCR |
|  |  |  |  | 18 Healthy | Asian | 27.60 ± 3.80 | - | - | 108.0 ± 13.60 | 81.00 ± 16.60 | 38.20 ± 1.10 | 38.20 ± 1.10 | No Information | 3145.0 ± 248.3 | FOXP3 | qPCR |
| Nguyen TA* | 2017 | USA | prospective observational cohort study | 16 PET | Caucasian 38% (6); Hispanic 19% (3); Black 19% (3); Asian 25% (4) p<0.0001 | 32.67±5.088 | - | 0 | - | - | 38.6 ± 0.6 | 38.6 ± 0.6 | No Information | 3231 ± 191 | CD4+ CD25+ CD127low | Flow cytometry |
|  |  |  |  | 30 Healthy | Caucasian 59% (17); Hispanic 28% (8); Black 10% (3); Asian 3% (2) p<0.0001 | 32.02±5.64 | - | 3% (1) | - | - | 39.1 ± 0.2 | 39.1 ± 0.2 | No Information | 3490 ± 84 | CD4+ CD25+ CD127low | Flow cytometry |
| Orlovic Vlaho M | 2020 | Bosnia and Herzegovina | case-control study | 15 PET | Caucasian | 28.50 ± 5.50 | 23.49 ± 2.49 | - | 175 ± 18.02 | 120 (110–140) | 34 (33–39) | 34 (33–39) | No Information | 1830 (1270–2200) | CD4+ CD25+ FOXP3+ | Immunofluorescence |
|  |  |  |  | 19 Healthy | Caucasian | 29.47 ± 4.26 | 25.92 ± 4.18 | - | 116 ± 6.94 | 75 (65–80) | 40 (37–41) | 40 (37–41) | No Information | 3250 (2250–4500) | CD4+ CD25+ FOXP3+ | Immunofluorescence |

***Study characteristics for meta-analysis comparing T regulatory cell numbers in the decidua of healthy pregnant women and women with pre-eclampsia toxaemia (PET); n=4.***

**Studies reporting both peripheral blood and decidual Treg analysis.*

***Supplementary Table 3***

| **Author** | **Year** | **Location** | **Study Design** | **Final N PTB /Healthy PBMCs** | **Ethnicity/**  **Race** | **Age group (years)** | **BMI** | **Smoking (%, n)** | **Blood pressure systolic (mmHg)** | **Blood pressure diastolic (mmHg)** | **Primigravidas (%, n)** | **Gestation at delivery (weeks)** | **Gestation at Treg analysis (weeks)** | **Steroids prior to isolation** | **Birth weight (grams)** | **Tregs marker** | **Method of analysis** |
| --- | --- | --- | --- | --- | --- | --- | --- | --- | --- | --- | --- | --- | --- | --- | --- | --- | --- |
| Xiong H | 2010 | China |  | 15 PTB | Asian | 26 ± 2 | - | - | - | - | - | 28–36 | 28–36 | No Information | 1781 ± 632 | CD4+  CD25+  FOXP3+ | Flow cytometry |
|  |  |  |  | 20 Healthy | Asian | 27 ± 2 | - | - | - | - | - | >37 | >37 | No Information | 3587 ± 281 | CD4+  CD25+  FOXP3+ | Flow cytometry |
| Koucky M | 2014 | Czech Republic | prospective study | 45 PTB | Caucasian | 32.5 | - | - | - | - | - | <37 | <37 | No Information | 2608.5±1016.32 | CD4+  CD25int/high  CD127low (number) | Flow cytometry |
|  |  |  |  | 15 Healthy | Caucasian | 32.5 | - | - | - | - | - | >37 | >37 | No Information | 2608.5±1016.32 | CD4+  CD25int/high  CD127low (number) | Flow cytometry |

***Study characteristics for meta-analysis comparing T regulatory cell numbers in the peripheral blood of healthy pregnant women and women who underwent preterm birth (PTB); n=2.***

*PBMCs peripheral blood mononuclear cells.*

***Supplementary Table 4***

| **Criteria** | **Acceptable**  **(star awarded):** | **Unacceptable**  **(star not awarded):** |
| --- | --- | --- |
| *Representativeness of exposed cohort* | Population-based | Hospital-based |
| *Selection of non-exposed cohort* | Same setting as exposed cohort | Different setting from exposed cohort |
| *Ascertainment of exposure* | Secure records or directly measured | Self-reported information |
| *Comparability* | Excluded or adjusted for prior outcome in analysis | No exclusion of prior outcome in previous pregnancy |
|  | Adjusted for age, race, smoking and interpregnancy interval | Did not adjust for age, race, smoking and interpregnancy interval |
| *Outcome of interest* | Secure records or directly measured | Self-reported information |
| *Adequacy of follow-up* | Adjusted for missing data or follow-up > 1 month. | No statement regarding missing data. No follow-up after birth. |

***Criteria for the Newcastle-Ottawa Scale regarding star allocation to assess quality of studies (out of a total of seven stars).***

***Supplementary Table 5***

| **Study ID** | | **Selection** | | | **Comparability*** | **Outcome** | | **Total (7**⋆**)** |
| --- | --- | --- | --- | --- | --- | --- | --- | --- |
| **Author** | **Year** | **Representativeness of exposed cohort (⋆)** | **Selection of non- exposed cohort (⋆)** | **Ascertainment of exposure (⋆)** | **(⋆⋆)** | **Assessment of outcome (⋆)** | **Adequacy of**  **follow up (⋆)** |  |
| *Schober L* | 2012 | 0 / 0 | (⋆) / (⋆) | (⋆) / (⋆) | 0 / 0 | (⋆) / (⋆) | 0 / 0 | 3⋆ / 3⋆ |
| ***Han X*** | 2019 | 0 / 0 | (⋆) / (⋆) | (⋆) / (⋆) | 0 / (⋆) | (⋆) / (⋆) | 0 / 0 | 3⋆ / 4⋆ |
| **Jianjun Z** | 2009 | 0 / 0 | (⋆) / (⋆) | (⋆) / (⋆) | (⋆) / (⋆⋆) | (⋆) / (⋆) | 0 / 0 | 4⋆ / 5⋆ |
| **Toldi G** | 2011 | 0 / 0 | (⋆) / (⋆) | (⋆) / (⋆) | (⋆) / (⋆⋆) | (⋆) / (⋆) | 0 / 0 | 4⋆ / 5⋆ |
| Darmochwal-Kolarz D | 2012 | 0 / 0 | (⋆) / (⋆) | (⋆) / (⋆) | (⋆) / (⋆) | (⋆) / (⋆) | 0 / 0 | 4⋆ / 4⋆ |
| **Toldi G** | 2012 | 0 / 0 | (⋆) / (⋆) | (⋆) / (⋆) | (⋆) / (⋆⋆) | (⋆) / (⋆) | 0 / 0 | 4⋆ / 5⋆ |
| Darmochwal-Kolarz D | 2012 | 0 / 0 | (⋆) / (⋆) | (⋆) / (⋆) | (⋆) / (⋆) | (⋆) / (⋆) | 0 / 0 | 4⋆ / 4⋆ |
| **Zeng B** | 2013 | 0 / 0 | (⋆) / (⋆) | (⋆) / (⋆) | (⋆) / (⋆⋆) | (⋆) / (⋆) | 0 / 0 | 4⋆ / 5⋆ |
| **Moreno-Eutimio MA** | 2014 | 0 / 0 | (⋆) / (⋆) | (⋆) / (⋆) | (⋆) / (⋆⋆) | (⋆) / (⋆) | 0 / 0 | 4⋆ / 5⋆ |
| Cao W | 2015 | 0 / 0 | (⋆) / (⋆) | (⋆) / (⋆) | 0 / 0 | (⋆) / (⋆) | 0 / 0 | 3⋆ / 3⋆ |
| Nagayama S | 2015 | 0 / 0 | (⋆) / (⋆) | (⋆) / (⋆) | (⋆⋆) / (⋆⋆) | (⋆) / (⋆) | 0 / 0 | 5⋆ / 5⋆ |
| Toldi G | 2015 | 0 / 0 | (⋆) / (⋆) | (⋆) / (⋆) | (⋆) / (⋆) | (⋆) / (⋆) | 0 / 0 | 4⋆ / 4⋆ |
| Wagner MI | 2016 | 0 / 0 | (⋆) / (⋆) | (⋆) / (⋆) | 0 / 0 | (⋆) / (⋆) | 0 / 0 | 3⋆ / 3⋆ |
| Vianna P | 2016 | 0 / 0 | (⋆) / (⋆) | (⋆) / (⋆) | (⋆⋆) / (⋆⋆) | (⋆) / (⋆) | (⋆) / (⋆) | 6⋆ / 6⋆ |
| Wang J | 2017 | 0 / 0 | (⋆) / (⋆) | (⋆) / (⋆) | 0 / 0 | (⋆) / (⋆) | 0 / 0 | 3⋆ / 3⋆ |
| Nguyen TA | 2017 | 0 / 0 | (⋆) / (⋆) | (⋆) / (⋆) | 0 / 0 | (⋆) / (⋆) | 0 / 0 | 3⋆ / 3⋆ |
| **Ribeiro VR** | 2017 | 0 / 0 | (⋆) / (⋆) | (⋆) / (⋆) | (⋆) / (⋆⋆) | (⋆) / (⋆) | 0 / 0 | 4⋆ / 5⋆ |
| **Yu J** | 2017 | 0 / 0 | (⋆) / (⋆) | (⋆) / (⋆) | (⋆) / (⋆⋆) | (⋆) / (⋆) | 0 / 0 | 4⋆ / 5⋆ |
| **Zhang Z** | 2017 | 0 / 0 | (⋆) / (⋆) | (⋆) / (⋆) | (⋆) / (⋆⋆) | (⋆) / (⋆) | 0 / 0 | 4⋆ / 5⋆ |
| Zare M | 2018 | 0 / 0 | (⋆) / (⋆) | (⋆) / (⋆) | (⋆) / (⋆) | (⋆) / (⋆) | 0 / 0 | 4⋆ / 4⋆ |
| Wang Y | 2018 | 0 / 0 | (⋆) / (⋆) | (⋆) / (⋆) | (⋆) / (⋆) | (⋆) / (⋆) | 0 / 0 | 4⋆ / 4⋆ |
| Salazar Garcia MD | 2018 | 0 / 0 | (⋆) / (⋆) | (⋆) / (⋆) | (⋆⋆) / (⋆⋆) | (⋆) / (⋆) | 0 / 0 | 5⋆ / 5⋆ |
| Chen J | 2018 | 0 / 0 | (⋆) / (⋆) | (⋆) / (⋆) | 0 / 0 | (⋆) / (⋆) | 0 / 0 | 3⋆ / 3⋆ |
| **Zhang Y** | 2018 | 0 / 0 | (⋆) / (⋆) | (⋆) / (⋆) | (⋆) / (⋆⋆) | (⋆) / (⋆) | 0 / 0 | 4⋆ / 5⋆ |
| **Daraei N** | 2019 | 0 / 0 | (⋆) / (⋆) | (⋆) / (⋆) | (⋆) / (⋆⋆) | (⋆) / (⋆) | 0 / 0 | 4⋆ / 5⋆ |
| Ding H | 2019 | 0 / 0 | (⋆) / (⋆) | (⋆) / (⋆) | (⋆) / (⋆) | (⋆) / (⋆) | 0 / 0 | 4⋆ / 4⋆ |
| Eghbal-Fard S | 2019 | 0 / 0 | (⋆) / (⋆) | (⋆) / (⋆) | 0 / 0 | (⋆) / (⋆) | 0 / 0 | 3⋆ / 3⋆ |
| Hu M | 2019 | 0 / 0 | (⋆) / (⋆) | (⋆) / (⋆) | 0 / 0 | (⋆) / (⋆) | 0 / 0 | 3⋆ / 3⋆ |
| **Jabalie G** | 2019 | 0 / 0 | (⋆) / (⋆) | (⋆) / (⋆) | (⋆) / (⋆⋆) | (⋆) / (⋆) | 0 / 0 | 4⋆/ 5⋆ |
| Li J | 2019 | 0 / 0 | (⋆) / (⋆) | (⋆) / (⋆) | (⋆) / (⋆) | (⋆) / (⋆) | 0 / 0 | 4⋆ / 4⋆ |
| Zare M | 2019 | 0 / 0 | (⋆) / (⋆) | (⋆) / (⋆) | (⋆) / (⋆) | (⋆) / (⋆) | 0 / 0 | 4⋆ / 4⋆ |
| Meggyes M | 2019 | 0 / 0 | (⋆) / (⋆) | (⋆) / (⋆) | (⋆) / (⋆) | (⋆) / (⋆) | 0 / 0 | 4⋆ / 4⋆ |
| **Xiong H** | 2010 | 0 / 0 | (⋆) / (⋆) | (⋆) / (⋆) | (⋆) / (⋆⋆) | (⋆) / (⋆) | 0 / 0 | 4⋆ / 5⋆ |
| Koucky M | 2014 | 0 / 0 | (⋆) / (⋆) | (⋆) / (⋆) | 0 / 0 | (⋆) / (⋆) | 0 / 0 | 3⋆ / 3⋆ |
| Liu X | 2011 | 0 / 0 | (⋆) / (⋆) | (⋆) / (⋆) | 0 / 0 | (⋆) / (⋆) | 0 / 0 | 3⋆ / 3⋆ |
| Orlovic Vlaho M | 2020 | 0 / 0 | (⋆) / (⋆) | (⋆) / (⋆) | 0 / 0 | (⋆) / (⋆) | 0 / 0 | 3⋆ / 3⋆ |

***Quality assessment for each study using a modified Newcastle-Ottawa scale broken down by dimension and rater (MP/KSR).***

**Comparability assessed as the following: one star rewarded if study excluded or adjusted for outcome in first pregnancy, another star rewarded if study adjusted for age, race, smoking and interpregnancy interval.*

*In bold studies with inter-rater scoring discrepancies.*

*In italics studies included in qualitative synthesis only.*
